# Transcriptional Dynamics of Genes Purportedly Involved in the Control of Meiosis, Carbohydrate, and Secondary Metabolism during Sporulation in *Ganoderma lucidum*

**DOI:** 10.3390/genes12040504

**Published:** 2021-03-29

**Authors:** Manjun Cai, Xiaowei Liang, Yuanchao Liu, Huiping Hu, Yizhen Xie, Shaodan Chen, Xiong Gao, Xiangmin Li, Chun Xiao, Diling Chen, Qingping Wu

**Affiliations:** 1 Guangdong Provincial Key Laboratory of Microbial Safety and Health, State Key Laboratory of Applied Microbiology Southern China, Institute of Microbiology, Guangdong Academy of Sciences, Guangzhou 510070, China; caimanjun4439@webmail.hzau.edu.cn (M.C.); liangxiaowei1@outlook.com (X.L.); liuyc1020@163.com (Y.L.); huhp@gdim.cn (H.H.); xieyizhen@126.com (Y.X.); shaodanchen@126.com (S.C.); gaoxiong881109@163.com (X.G.); xiangmin227@163.com (X.L.); xiaochun960@hotmail.com (C.X.); diling1983@163.com (D.C.); 2 Guangdong Yuewei Edible Fungi Technology Co. Ltd., Guangzhou 510663, China

**Keywords:** *Ganoderma lucidum*, sporulation, transcription regulation, energy source, meiotic process

## Abstract

*Ganoderma lucidum* spores (GLS), the mature germ cells ejected from the abaxial side of the pileus, have diverse pharmacological effects. However, the genetic regulation of sporulation in this fungus remains unknown. Here, samples corresponding to the abaxial side of the pileus were collected from strain YW-1 at three sequential developmental stages and were then subjected to a transcriptome assay. We identified 1598 differentially expressed genes (DEGs) and found that the genes related to carbohydrate metabolism were strongly expressed during spore morphogenesis. In particular, genes involved in trehalose and malate synthesis were upregulated, implying the accumulation of specific carbohydrates in mature *G. lucidum* spores. Furthermore, the expression of genes involved in triterpenoid and ergosterol biosynthesis was high in the young fruiting body but gradually decreased with sporulation. Finally, spore development-related regulatory pathways were explored by analyzing the DNA binding motifs of 24 transcription factors that are considered to participate in the control of sporulation. Our results provide a dataset of dynamic gene expression during sporulation in *G. lucidum*. They also shed light on genes potentially involved in transcriptional regulation of the meiotic process, metabolism pathways in energy provision, and ganoderic acids and ergosterol biosynthesis.

## 1. Introduction

*Ganoderma lucidum* (Leyss. ex Fr.) Karst, a well-known medicinal mushroom, has been used in traditional Chinese medicine for centuries owing to its medicinal properties [1]. *Ganoderma lucidum* spores (GLS) are the mature germ cells of *G. lucidum* [2] and have been shown to contain a variety of bioactive components similar to other parts of *G. lucidum*, including triterpenoids, polysaccharides, nucleosides, and alkaloids [3,4]. In particular, GLS were enriched with five betaines, indicating that GLS can be used to develop drugs for the treatment of liver disease and diabetes. Furthermore, compared to the fermentation broth, mycelium, and fruiting body, GLS contain 28 unique metabolites, including azathioprine, geranylacetone, turmeronol A, and remikiren [4]. With the advancement of technology for breaking the spore wall and extracting bioactive compounds from spores, GLS have been widely used in immunoregulation, antitumor treatment, neuroprotection, hepatoprotection, antioxidation, anti-radiation, and anti-mutation [2]. Therefore, the GLS industry is flourishing and has great economic potential [5,6]. 

Although the pharmacological effects have been demonstrated, the genes and metabolic pathways involved in the crucial stages of sporulation in *G. lucidum* are still unknown. With the completion of genome sequencing of *G. lucidum* [7] and the development of genetic tools, including gene silencing systems [8] and dual sgRNA-directed gene deletion systems [9], molecular genetics research in this species is increasing exponentially [10,11,12,13]. Since forward genetic dissection of favorable traits is a time-consuming and arduous task in basidiomycete mushrooms, comprehensive analysis using omics technologies has become a feasible option.

RNA sequencing (RNA-seq) has proven to be a powerful tool for unravelling complex biological processes and has been successfully utilized in fungi. To identify genes involved in the loss of quality of *Lentinula edodes* postharvest fruiting bodies, transcriptome analysis was performed, and it was found that several cell wall- and chitin-related genes were expressed after harvest. These genes are potential targets for the future breeding of strains that remain fresh for longer periods than the present strains [14]. Primordia development defect1 (PDD1) is a novel transcription factor with regulatory function in *Flammulina velutipes* basidioma development. RNA sequencing analysis has revealed that knockdown of the *pdd1* gene causes the downregulation of several genes related to primordium formation [15]. Transcriptome analysis of different developmental stages of peridioles identified transcription factors (TFs) that may play a role in this regulation [16]. Previous studies on comparative transcriptome analysis have identified candidate genes involved in mycelium browning [17,18] and revealed a high rate of divergence in developmental gene expression as well as several genes with conserved expression patterns [19,20]. These studies indicate that RNA-seq provides an effective way to explore the underlying regulatory networks of favorable traits in fungi. 

Several studies on *G. lucidum* have been conducted to elucidate changes in gene transcription in response to stress conditions [21,22] or at different developmental stages, mainly in the mycelium, primordia, and fruiting body stages [7,23,24]. However, the genes involved in *G. lucidum* spore morphogenesis remain unknown. In the present study, developmental transcriptome analyses of three stages of sporulation in *G. lucidum* were performed using RNA-seq. We identified several TFs, functional genes, and major metabolic pathways that might be associated with spore morphogenesis and secondary metabolism in *G. lucidum*. These data help expand our understanding of the transcriptional landscape of basidiosporogenesis in *G. lucidum* and also provide a valuable source for elucidating the genetic regulatory networks underlying *G. lucidum* spore development.

## 2. Materials and Methods 

### 2.1. Sample Collection at Differential Stages

**Figure 1:**
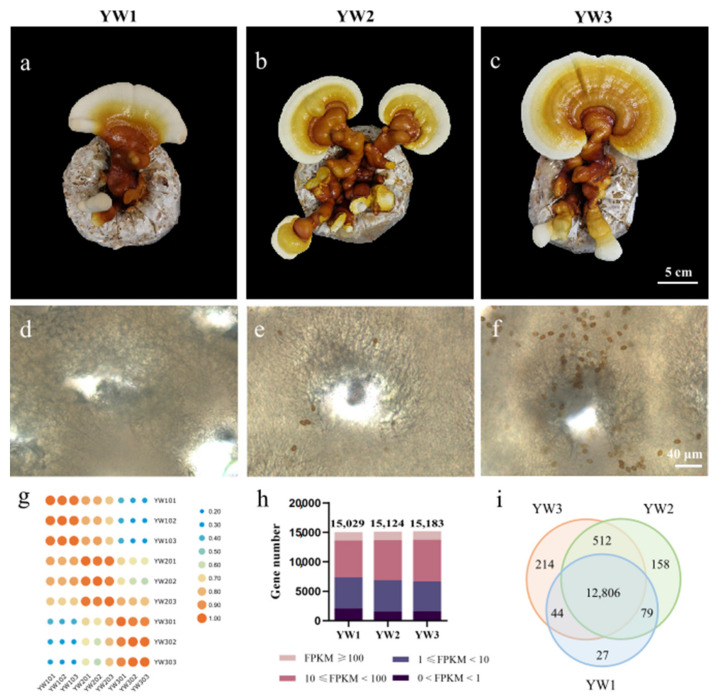
Summary of transcriptome sequence datasets. (**a**–**f**) Developmental comparisons of pileus (**a**–**c**) and cross section of gill (**d**–**f**) at three spore developmental stages of YW-1 strain. Young fruiting body before sporulation (**a**,**d**), initial stage of sporulation (**b**,**e**), and nearly mature fruiting body with lots of spores (**c**,**f**) are referred to as YW1, YW2, and YW3, respectively. (**g**) Correlation among transcriptome datasets. Pearson’s correlation coefficient r was used to assess the reliability of every two RNA sequencing (RNA-Seq) libraries. A score of near 1 between biological replicates indicates faithful replication, while a much lower score between different developmental stages indicates globally different. (**h**) Distribution diagram shows the number of genes with different fragments per kilobase of transcript per million fragments mapped (FPKM) values across the three developmental stages. (**i**) Venn diagram shows the number of stage-specific expressed genes at each developmental stage and the shared expression genes between stages.

*Ganoderma lucidum* dikaryotic strain YW-1 from the Institute of Microbiology Guangdong Academy of Sciences was selected for our study because of its outstanding performance during fruiting body development. After 20 days of growth on sorghum medium at 25 °C, mycelia were inoculated onto the substrate packed in heat-sealed cultivation bags with microfilter windows and cultured in the dark at 25 °C for 1 month. For fruiting body growth, the bags were cultured in a room with 10 h of illumination and 30 min of ventilation at 26 °C. In our study, *G. lucidum* spore development was divided into three stages, YW1, YW2, and YW3, according to their fruiting body morphology and spore number. Fruiting bodies in YW1 are the youngest with a large area of white edge, without pores on the abaxial side of the pileus, and have no spores. Those in YW2 begin to show obvious pores in the center of the abaxial side of the pileus and have 8–10 spores near every pore, as shown in Figure 1b. Mature fruiting bodies with numerous spores were assigned as YW3.

Abaxial sides of the pileus from at least three independent bags with the same developmental stage were selected and quickly frozen in liquid nitrogen. Three replicate samples were prepared for each developmental stage. All samples were stored at −80 °C prior to RNA isolation.

### 2.2. Total RNA Extraction and Sequencing

Total RNA was extracted from the samples using the TRIzol Kit (Invitrogen, Carlsbad, CA, USA), according to the manufacturer’s instructions. RNA concentration was assessed using Nanodrop 2.0 (Thermo Fisher Scientific, Waltham, MA, USA) and Agilent 2100 Bioanalyzer (Agilent, Santa Clara, CA, USA), and RNA integrity was assessed using gel electrophoresis. The library was constructed by enrichment of eukaryotic mRNAs using oligo-dT with magnetic beads, and mRNAs were randomly interrupted and reverse transcribed into cDNA using random oligonucleotides. Double-stranded cDNA was purified, followed by terminal repair and the addition of a tail and sequenced connector. Next, a cDNA library was obtained using PCR enrichment. Finally, Illumina HiSeq mRNA sequencing was used for high-throughput sequencing with paired-end 150-bp reads (BioMarker Technology, Beijing, China).

### 2.3. Transcriptome Assembly and Gene Annotation

All raw reads were deposited into the Sequence Read Archive database (accession number: PRJNA704770) and trimmed for low-quality reads, Illumina adapters, and sequences shorter than 15 nucleotides. Next, clean reads were mapped to the *Ganoderma lucidum* genome using HISAT2 [25] and String Tie [26]. Fragments per kilobase of transcript per million fragments mapped (FPKM) was used to quantify the expression of each gene [26]. Differential expression analysis between pairs of samples was performed using the DESeq2 package [27] to identify differentially expressed genes (DEGs), and the adjusted *p*-values were calculated using the Benjamini-Hochberg method to control the false discovery rate (FDR). FDR < 0.01 and fold change ≥ 2 were set to screen DEGs. The DEG sequences were blasted with GO (Gene Ontology), COG (Clusters of Orthologous Groups of proteins), KEGG (Kyoto Encyclopedia of Genes and Genomes), KOG (Eukaryotic Orthologous Groups of proteins), Pfam, Swiss-Prot, eggNOG (evolutionary genealogy of genes: Non-supervised Orthologous Groups), and NR (Non-Redundant Protein Sequence Database) databases using BLAST software.

### 2.4. Transcriptional Regulatory Pathway Prediction

*Cis*-acting elements in the promoter regions of 835 developmentally regulated genes were predicted using PlantCARE [28]. The motifs found in every gene were summarized in MS Excel software and further processed to identify possible transcriptional regulator binding sequences. A total of 106 DEGs containing GATA-binding sequences were selected. These DEG sequences were blasted with GO, COG, KEGG, KOG, Pfam, Swiss-Prot, eggNOG, and NR databases using BLAST software to explore their possible functions. 

### 2.5. Quantitative Real-Time PCR (qRT-PCR) Validation

The extracted total RNA from all samples subjected to transcriptome analysis was used for qRT-PCR. Single-stranded cDNA was synthesized using HiScript^®^ II Q RT SuperMix for qRT-PCR (+gDNA wiper) (Vazyme Biotech, Nanjing, China), according to the manufacturer’s instructions. Ten genes were selected to verify the reliability of RNA-seq data. The gene-specific primers used for gene quantification were designed using Primer3 Input. qRT-PCR was performed using gene-specific primers (Table S1) in a 20 µL reaction with a 2 × AceQ^®^ qPCR SYBR^®^ Green Master Mix (Vazyme Biotech, Nanjing, China) under the following conditions: initial denaturation at 95 °C for 2 min, followed by 40 cycles at 95 °C for 15 s and at 60 °C for 20 s. qRT-PCR was performed using the Applied Biosystems ABI 7500 (Applied Biosystems, Foster City, CA, USA) with three biological and technical repeats. The internal reference gene 18 S was used to normalize the expression data. Relative expression levels were calculated according to the 2−∆∆CT (CT, cycle threshold) method [29].

## 3. Results

### 3.1. Differences between Sporulation Stages were Evident at the Transcriptional Level

To obtain an overview of the *G. lucidum* gene expression profiles during sporulation, triplicate samples from the abaxial side of the pileus at three different developmental stages, YW1 (newly formed fruiting body without spores) (Figure 1a,d), YW2 (developing fruiting body with eight to ten spores near every pore) (Figure 1b,e), and YW3 (nearly mature fruiting body with numerous spores) (Figure 1c,f), were collected for transcriptome sequencing. After filtering for adaptor sequences and low-quality reads, approximately 86% of the reads could be mapped to the *G. lucidum* genome (Table S2). A subset of 375 genes that were not found in the monokaryotic strain G.260125-1 were identified in the YW-1 strain (Table S3). Gene expression levels among replicates for each stage exhibited a high Pearson’s correlation coefficient value, while the differences between stages were evident (Figure 1g), indicating that these three sporulation stages were obviously different at the transcriptional level and the biological replicates had good reproducibility. 

As shown in Figure 1h, the majority of genes (44.3%) were moderately expressed, with FPKM values ranging from 10 to 100, followed by the genes (34.5%) with FPKM values between 1 and 10. Only 9.5% of the genes were highly expressed with FPKM values > 100. Genes with an FPKM value < 1 were considered silent, which accounted for approximately 11% of the genes at each developmental stage. Among the detected genes, 12,806 genes were expressed across these three stages, while 27, 158, and 214 genes were specific to the YW1, YW2, and YW3 stages, respectively (Figure 1i). Of all the transcripts, 12,109 genes could be annotated using a sequence similarity search in public protein databases (Table 1). Even though more than 99% of the genes could be annotated in the NR database, most of them were hypothetical proteins annotated in the *Ganoderma sinense* ZZ0214-1 genome and could not provide any valuable information. Fortunately, 7207, 5712, 5502, and 4985 genes were matched in Pfam annotation, Swiss-Prot annotation, GO annotation, and KOG annotation, respectively (Table 1).

### 3.2. Functional Classification of Differentially Expressed Genes across Three Developmental Stages

**Figure 2:**
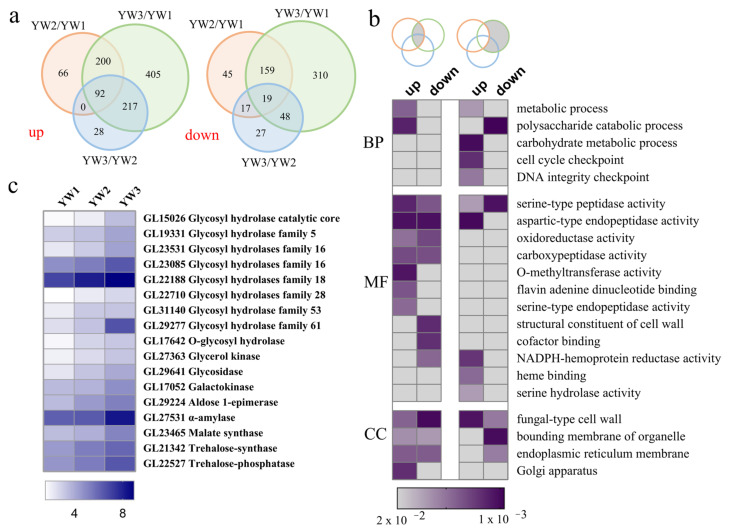
Gene Ontology (GO) functional enrichment of differentially expressed genes. (**a**) Venn diagrams show the number of differentially expressed genes (DEGs) between different developmental stages and the number of shared DEGs. The left Venn diagram indicates the upregulated genes, and the right one represents the downregulated genes. (**b**) GO enrichment for shared DEGs between different comparisons. Venn diagrams represent DEGs shared among different comparisons (from left to right): DEGs in YW2/YW1 and YW3/YW1; DEGs in YW3/YW1, but not in YW2/YW1. BP: biological process; MF: molecular function; CC: cellular component. The GO terms with *p* values < 0.01 are shown in color, otherwise, they are shown in grey. (**c**) Expression profiles of DEGs in carbohydrate metabolic process. The square represents the log_2_(FPKM + 1) ratio of the expression level in different developmental stages.

By comparing the relative abundance of transcripts at three stages, a total of 1598 DEGs were detected (Table S4) under FDR < 0.01, and fold change (FC) ≥ 2. Among the three stages, 1450 genes showed differences in expression between YW3 and YW1, which accounted for 90.74% of all DEGs, concurrent with the developmental and physiological status of these two stages. A total of 581 DEGs were found between YW2 and YW1, and 431 DEGs were found between YW3 and YW2, suggesting that the difference between the two adjacent developmental stages was less obvious. Meanwhile, the difference between the stage before sporulation (YW1) and mature stage (YW3) was significant (Figure 2a), and differences in gene expression levels were consistent with the developmental stages. GO, COG, KEGG, KOG, Pfam, Swiss-Prot, eggNOG, and NR databases were used to annotate the functions of the DEGs (Table S4). Approximately 88% of the DEGs were annotated using databases listed above (Table 2).

To evaluate the potential roles of these DEGs, GO enrichment analysis was performed. Considered that spore morphogenesis occurs at the YW2 and YW3 stages, thus the shared DEGs between YW2/YW1 and YW3/YW1 might be involved in the initiation of spore morphogenesis. In addition, spore development occurs at the YW3 stage, thus, DEGs between YW3 and YW1 might be involved in the control of spore development (Figure 2b). As shown in Figure 2b, a functional shift during spore morphogenesis and development was observed in both up- and down-regulated genes. Notably, DEGs involved in carbohydrate metabolic process was upregulated at the later stage of sporulation (Figure 2b). Half of these DEGs encoded glycosyl hydrolases (GH), which primarily hydrolyze polysaccharides (Figure 2c). In addition to genes involved in polysaccharide hydrolysis, genes involved in carbohydrate synthesis, including malate and trehalose synthase genes, were also highly expressed at the YW3 stage relative to the YW1 stage (Figure 2c). These results suggest that at the YW3 stage, carbohydrates might be hydrolyzed to provide energy for the rapid growth of organisms, and the specific carbohydrates, such as malate and trehalose, are inclined to accumulate and serve as energy sources for further development.

**Figure 3:**
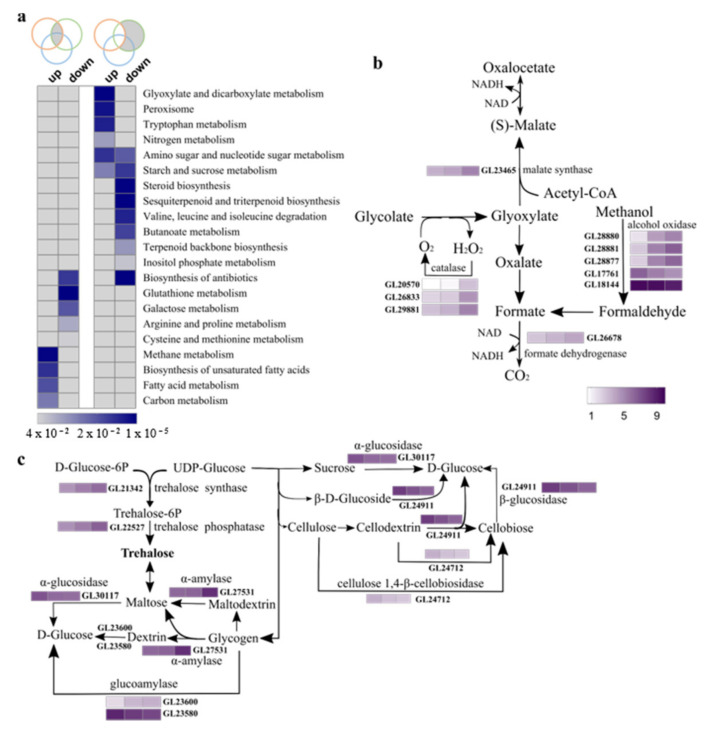
Kyoto Encyclopedia of Genes and Genomes (KEGG) pathway enrichment of differentially expressed genes. (**a**) KEGG enrichment for shared DEGs between different comparisons. Venn diagrams represent DEGs shared among different comparisons. Significantly enriched pathways with *p* < 0.05 are shown. (**b**) Expression of genes in the carbon metabolic pathway. (**c**) Expression of genes in the starch and sucrose metabolism pathway. The square represents the log_2_(FPKM + 1) ratio of the expression level in different developmental stages.

To further elucidate the biological pathways of the DEGs, KEGG pathway analysis was performed. As shown in Figure 3a, a change in pathway enrichment was observed for both up- and down-regulated genes. It was obvious that DEGs predictably involved in carbohydrate metabolisms, including methane, carbon, glyoxylate and dicarboxylate, and starch and sucrose metabolism, were significantly upregulated, which is consistent with GO enrichment. Furthermore, we found that DEGs involved in methane (ko00680) as well as glyoxylate and dicarboxylate metabolism (ko00630) also participated in carbon metabolism (ko01200), especially the malate synthase and formate dehydrogenase encoding genes, *GL23465* and *GL26678* (Figure 3b). This suggests that malate accumulation and NADH production increased during sporulation. In addition, genes involved in starch and sucrose metabolism (ko00500) were also significantly enriched. Meanwhile, expression of *GL21342* and *GL22527*, genes coding for key enzymes in trehalose synthesis, were constantly increased; the gene expression profile in glycogen catabolism indicated that glycogen was catabolized into maltose but not D-glucose (Figure 3c). The expression dynamics of these genes might reflect the accumulation of trehalose and maltose at the YW3 stage. In contrast, the expression levels of three glycosidase-encoding genes were reduced (Figure 3c). We inferred that NADH and intermediate metabolites from carbon metabolism, especially from glyoxylate and dicarboxylate metabolism, were required for fruiting body growth and spore development at the YW3 stage. 

**Figure 4:**
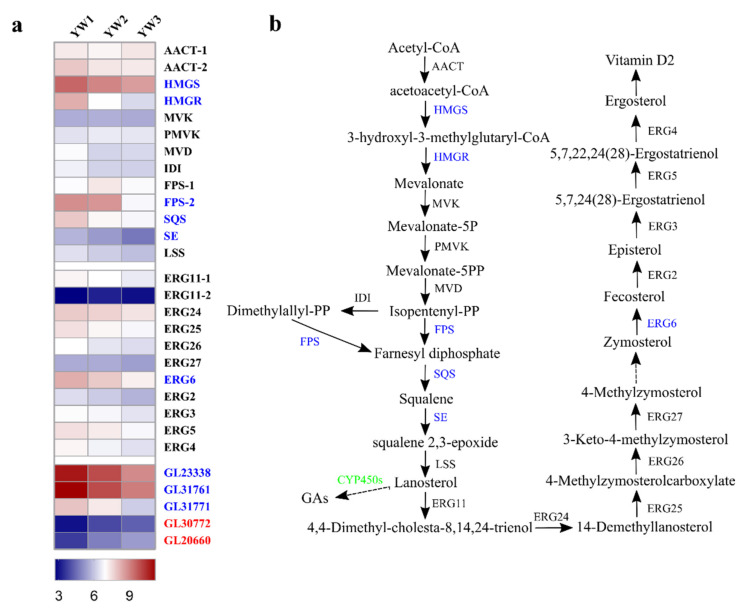
DEGs predictably involved in triterpenoid and ergosterol biosynthesis pathways. (**a**) Heatmap shows the expression level of the genes coding for key enzymes, which are predictably involved in triterpenoid and ergosterol biosynthesis pathways. The square represents the log_2_ ratio of the expression level in different developmental stages. Red, upregulated; blue, downregulated. (**b**) A schematic of triterpenoid and ergosterol biosynthesis pathways in *Ganoderma lucidum*.

Secondary metabolites are one of the major groups of therapeutic compounds in *G. lucidum*. As shown in Figure 3a, DEGs involved in multiple secondary metabolite biosynthesis pathways, including steroid, sesquiterpenoid, and triterpenoid, and terpenoid backbone biosynthesis, were downregulated at the YW3 stage when compared to the YW1 stage. We found that DEGs involved in these pathways also participated in the control of ganoderic acids (GAs) and ergosterol production. GAs and ergosterol produced by *G. lucidum* exhibit pharmacological activity [30]. Previous studies have identified 24 key genes involved in the biosynthesis of these bioactive compounds [31]. The relative expression levels of these 24 genes are shown in Figure 4a. Mevalonate is the only precursor of triterpenoids, and 3-hydroxy-3-methylglutaryl CoA reductase (HMGR) is the rate-limiting enzyme that catalyzes 3-hydroxy-3-methylglutaryl CoA into mevalonate [31]. We found that expression of the HMGR-encoding gene, *GL24088*, decreased sharply at both the YW2 and YW3 stages when compared to the YW1 stage. Farnesyl-diphosphate synthase (FPS) is encoded by two genes, *FPS-1* and *FPS-2*. Although *FPS-1* was transcribed stably during sporulation, *FPS-2* was highly expressed in YW1 and YW2 stages but decreased sharply in YW3 stage, and its expression level was higher than that of *FPS-1*. The expression of genes encoding 3-hydroxy-3-methylglutaryl CoA synthase (HMGS), squalene synthase (SQS), and squalene monooxygenase (SE) in ganoderic acid biosynthesis were significantly decreased at the later stage of sporulation (Figure 4). These results suggest that lanosterol, the common cyclic intermediate of triterpenoids and ergosterol, might be decreased at the YW3 stage when compared to the YW1 stage. 

Lanosterol is a key precursor of multiple metabolites, including GAs and ergosterol. Cytochrome P450s (CYP450s) play an essential role in lanosterol oxidation. The expression profiles of 78 CYP450 genes are highly correlated with that of the lanosterol synthase (LSS) gene and positively correlated with triterpenoid content during the development process [7]. In our study, 5 of 78 CYP450 genes (*GL30772, GL20660, GL31771, GL31761*, *and GL23338*) were differentially expressed during sporulation (Figure 4a). We found that both *GL30772* and *GL20660* showed an expression pattern opposite to *LSS* expression, and the remaining three genes were co-expressed with *LSS*. With respect to ergosterol biosynthesis, only *ERG6* was significantly downregulated in YW3 compared to YW1 and YW2. These results could indicate that young fruiting bodies might produce more GAs and ergosterol than those in the mature stage. Furthermore, two fumagillin β-trans-bergamotene synthases encoding genes, *GL18506* and *GL24809*, which belong to the UbiA prenyltransferase family, were differentially expressed (Table S4). It has been reported that UbiA prenyltransferase domain-containing protein-1 (UBIAD1) utilizes geranylgeranyl pyrophosphate (GGpp) to synthesize vitamin K2, which is the branched pathway of farnesyl diphosphate [32]. This finding suggests that the downregulation of the lanosterol synthetic pathway might have resulted from the upregulation of the branched pathway of farnesyl diphosphate.

### 3.3. Developmentally Regulated Transcriptional Regulators and Their Possible Regulatory Pathways

**Figure 5:**
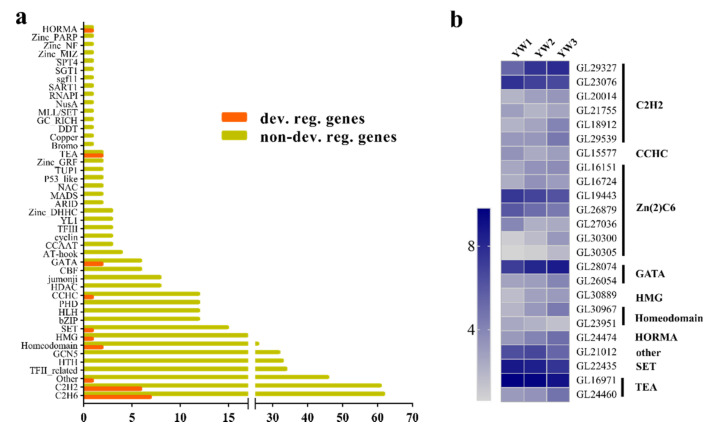
Expression of genes coding for transcriptional regulators. (**a**) Transcriptional regulator family distribution and the proportions of developmentally regulated (orange) versus non-regulated (yellow) genes. (**b**) Heatmap of developmentally regulated transcription factor (TF)-coding genes. The square represents log_2_(FPKM + 1) ratio of the transcript abundance change in different stages.

More than 600 genes coding for transcriptional regulators have been identified in the *G. lucidum* genome [7]. In this study, 476 of these 600 regulators had FPKM values > 1 (Table S5). During spore development, 24 transcripts coding for transcriptional regulators were found to be differentially regulated (Figure 5a). Of these, 15 genes belonged to the zinc finger family, including six C2H2-containing TFs, seven Zn2Cys6-containing TFs, two GATA-containing TFs, and one CCHC-containing TF (Figure 5b). Four genes were homologous with transcriptional regulators that are considered to play a role in sporulation (Table S6). For example, GL24474 is homologous to *Saccharomyces cerevisiae* HOP1, a meiosis-specific component [33,34]. *GL16971* encodes a TEA-containing protein and shows high similarity with the conidiophore development regulator AbaA [35,36]. CON7 has been proven to control spore morphogenesis in the rice blast fungus *Magnaporthe grisea* by regulating the transcription of several genes, which may encode factors determining cell wall structure or function [37,38]; the homolog of CON7 in *G. lucidum* was GL21755, which was differentially regulated during sporulation. Finally, the homolog of GL23076 is ZFP1 in *Cryptococcus neoformans*, and ZFP1 is also essential for fungal sexual reproduction because basidiospore production was blocked in bilateral mating between *zfp1*Δ mutants or strains overexpressing *ZFP1* [39].

**Figure 6:**
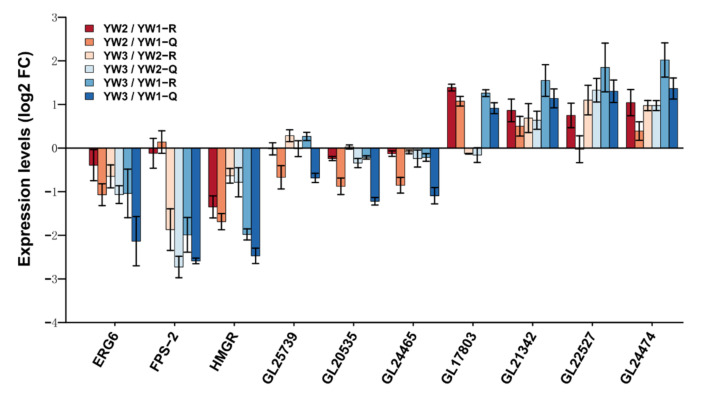
Expression level of selected genes in different developmental stages analyzed by qRT-PCR. qRT-PCR verification of the expression of seven differentially expressed genes (DEGs) and three non-differentially expressed genes (*GL25739*, *GL20535,* and *GL24465*) in three stages. Q: qRT-PCR; R: RNA-seq.

To further explore the regulatory pathway of sporulation, a total of 835 developmentally regulated genes, which can be annotated by at least one database, were identified, and the *cis*-acting elements in their promoter regions were predicted. In total, we found 116 GATA-binding sequences ([A/T]GATA[A/G]) (Table S7) in the promoters of 106 DEGs (Table S8), indicating that two differentially expressed GATA genes (*GL28074* and *GL26054*) might regulate the spatiotemporal expression patterns of these 106 DEGs by binding to the [A/T]GATA[A/G] sequence. However, the binding sequences of C2H2 and Zn(2)C6 were not found. Importantly, two of these 106 DEGs were sporulation-associated genes (*GL17992* and *GL17803*). *GL17992* encodes a meiotic recombination protein, and its homolog is SPO11/REC12. Recombination between homologous chromosomes during meiosis is an essential process, and *Spo11* is one of the key genes involved in this process. In addition, *Spo11* and its function are conserved from yeast to humans, and its mutation results in partial or complete sterility [40]. The homolog of GL17803 in *Schizosaccharomyces pombe* is the RNA-binding protein Mei2, which is crucial for initiating pre-meiotic DNA synthesis and meiosis [41]. *GL17803* was upregulated at the YW2 and YW3 stages compared to the YW1 stage (Figure 6). We speculated that GL17803 might also function in the pre-meiotic S-phase and was repressed during vegetative growth.

### 3.4. qRT-PCR Validation of the Reliability of the RNA-Seq Analysis 

To validate the transcriptome analysis, 10 genes, including 7 DEGs and 3 genes showing a constant expression pattern, were selected to assess the expression dynamics during sporulation by qRT-PCR. The expression trends of the eight genes in different comparisons were consistent with the RNA-Seq data (Figure 6). Two 1,3-β-glucan synthase encoding genes, *GL20535* and *GL24465*, were not differentially expressed in the RNA-seq analysis but were downregulated at the YW2 and YW3 stages as compared to the YW1 stage. In addition, compared to the YW1 stage, *ERG6* was downregulated significantly at the YW3 stage in transcriptome analysis, but was downregulated at the YW2 and YW3 stages by qRT-PCR.

## 4. Discussions and Conclusions

*G. lucidum* has been used in traditional Chinese medicine for thousands of years [42]. *Ganoderma lucidum* spores (GLS) are the mature germ cells of *G. lucidum*. Apart from having active components similar to fruiting bodies, they also contain several specific metabolites [4]. Current studies focus on the pharmacological aspects; however, the understanding of the basic biology of GLS is very limited. Developmental transcriptomics can help expand our understanding of the transcriptional landscape of basidiosporogenesis in *G. lucidum*. Here, we provided transcriptomic data from three developmental stages of GLS. Of particular focus were the spatiotemporal expression patterns of genes in representative carbohydrate metabolic pathways, meiosis, and transcriptional regulation, which are valuable targets for spore yield breeding. 

### 4.1. Several Developmentally Regulated Transcription Regulators Contribute to Sporulation

Transcriptional factors play a central role in orchestrating spatiotemporally precise gene expression programs, which are essential for the proper control of growth and development in all organisms [43,44,45]. In *Neurospora crassa*, 273 genes encoding transcription factors have been knocked out by high-throughput gene knockout procedures [46,47]; 19% of mutants have defective sexual cycles and 89% of the sexual mutants are partially or completely blocked in ascospore production. Mutants with a sexual cycle phenotype are mostly attributed to the knockout of genes encoding C2H2 and Zn2Cys6 [47]. Zinc finger proteins are reportedly involved in many cellular processes, including the regulation of conidial development and sexual sporulation [47,48]. In *Cryptococcus neoformans*, the involvement of 37 TFs in the mating stages has been proven by homologous recombination [49]. In the present study, we found that 24 transcriptional regulators were developmentally regulated during sporulation in *G. lucidum*, including seven Zn2Cys6, six C2H2, two GATA/Homeodomain/TEA, and one CCHC/HMG/HORMA/SET/Other. Through sequence similarity search, we found that the homologs of GL23076 (ZFP1, C2H2) [39], GL21755 (CON7, C2H2) [37], GL24474 (HOP1, HORMA) [33,34,50], and GL16971 (AbaA, TEA) [35,36] have been cloned and characterized as essential regulators of fungal spore morphogenesis. The expression dynamics were consistent with their putative function; therefore, we inferred that these four differentially expressed genes might be involved in sporulation in *G. lucidum*, although their biological functions are yet to be determined.

### 4.2. Meiosis-Related Genes Are Necessary for Sporulation

The life cycles of eukaryotes alternate between haploid and diploid phases, which are initiated by meiosis and gamete fusion, respectively [51]. Remarkably, in both ascomycete and basidiomycete fungi, spore morphogenesis is generated from diploid nuclei that undergo two rounds of meiosis [52,53], which implies that the meiotic process is necessary for sporulation. Disruption of genes involved in meiosis results in defective spores; examples include involvement in chromosome condensation [54], synapsis [55], recombination [40,56,57], homolog pairing [55], meiotic spindle formation, and meiotic programmed cell death [58]. 

Based on the comprehensive exploration of the *cis*-acting regulatory element on the promoter of all annotated DEGs, we found that *GL17803* and *GL17792*, which encode the apparent homologs of *Schizosaccharomyces pombe* Mei2 and Rec12/Spo11 in *G. lucidum*, might be involved in spore morphogenesis. The *Mei2* gene that encodes an RNA-binding protein (RBP) in premeiotic DNA synthesis and meiosis in yeasts and animals [59], which plays pivotal roles in regulating meiosis-specific gene expression [60], is degraded via the ubiquitin-proteasome pathway in a phosphorylation-dependent manner by Pat1 kinase. Mutants of Mei2, which cannot be phosphorylated and degraded by the proteasome, initiate meiosis without nitrogen starvation [61,62]. Compared to YW1, *GL17803* was upregulated in YW2 and YW3, which was consistent with the expression dynamics of *Mei2*. Further functional research, such as gene knockout, is required to prove the involvement of GL17803 in the meiotic process. 

DNA double-strand breaks (DSBs) are the initiators of most meiotic homologous recombination events; Spo11 functions in this process by a type II DNA topoisomerase-like activity and its function has been conserved through evolution [40]. Further analysis revealed that both N-terminal and C-terminal non-conserved residues of Spo11 are essential for the function of the protein [63], but the N-terminal and C-terminal residues of GL17792 were different from those in Spo11, possibly for interaction with other meiotic DSB enzymes. In addition, five other DEGs were upregulated at the YW2 or YW3 stages compared to the YW1 stage, which were predicted to participate in meiotic processes such as replication, recombination, and repair (Table S9). Since the mechanisms and molecular machinery associated with meiosis are well-conserved within most eukaryotes, these genes might also participate in the meiotic process in *G. lucidum*.

### 4.3. Carbohydrate Metabolites May Play Dual Roles in Sporulation

We found that the glyoxylate cycle, a metabolic pathway that permits the use of two-carbon compounds as carbon sources and participates in the conversion of stored lipids to carbohydrates, was upregulated during spore development (Figure 3c). The key enzyme in this route, malate synthase (Mls), which converts glyoxylate and acetyl-CoA to malate, was upregulated at the YW2 and YW3 stages (Figure 3c). The activation of the glyoxylate cycle has also been observed during *Pisolithus microcarpus* basidiospore formation [16], and the loss of Mls function has resulted in germinating defects of spores [64,65,66]. These results indicate that the glyoxylate cycle is essential for sporulation and spore germination. In addition to the glyoxylate cycle, the methane metabolism pathway was also regulated during sporulation, and the upregulated formate dehydrogenase might contribute to NADH generation (Figure 3c). Taken together, the activation of carbon metabolism, including the glyoxylate cycle and methane metabolism, might be important for energy provision for GLS development.

In fungi, trehalose functions as a reserve carbohydrate that accumulates in the reproductive stages and is mobilized during germination [67]. The synthesis of trehalose during sporulation and the resulting accumulation of trehalose in spores has been observed in *Aspergillus fumigatus* [68]. Two putative trehalose synthesis genes, *GL21342* (trehalose 6-phosphate synthase gene) and *GL22527* (trehalose 6-phosphate phosphatase gene), were significantly upregulated during the spore morphogenesis. We inferred that more trehalose and trehalose-6-phosphate (T6P) accumulated at the YW2 and YW3 stages, which might serve as storage compounds or stress protectants to enhance spore resistance under extreme environmental conditions. 

Studies have shown that trehalose also functions as a metabolic signal for determining cell fate decisions during sexual development, and trehalose levels are a readout for glucose availability, and spore formation is favored when glucose becomes limiting [69]. In plants, carbohydrates are thought to play a crucial role in the regulation of flowering, and T6P has been suggested to function as a proxy for carbohydrate status and is required for the timely initiation of flowering [70]. Recently, T6P has been shown to act as a signaling sugar that governs auxin levels during the seed-filling process [71]. The other carbohydrate storage molecule, glycogen, also drives cell fate decisions between chlamydospore formation and sporulation [72]. These results indicate that glucose, glycogen, trehalose, and T6P from the starch and sucrose metabolism pathway acted not only as energy sources, but also as endogenous signals to ensure a timely transition between developmental stages. Therefore, we could not exclude the possibility that these metabolites might have dual roles during GLS development, which requires further research. 

### 4.4. Changes in Expression of Genes That Are Predictably Involved in Bioactive Metabolites Biosynthesis during Sporulation 

*G. lucidum* polysaccharide (GLPS), a major bioactive component, has been developed into modern medicines with pharmacological properties [73]. Present studies focus on the pharmacological effects, structural features, and properties of GLPS, while knowledge about its accumulation pattern during development is limited. In our study, based on the dynamics of starch and sucrose metabolism (Figure 3c), we speculated that developing fruiting bodies with numerous spores contained more trehalose, maltose, and sucrose, and DEGs in this pathway could be regulated to influence carbohydrate components. Previous studies have shown that the total soluble polysaccharide content decreases after primordium develops into a fruiting body in strain G0119 [74]. In our study, we speculated that the polysaccharide composition varied during the development of the fruiting body. 

It has been reported that triterpenoid content is clearly reduced from primordia to mature fruiting bodies [7]; however, fruiting bodies have been collected for commercial use for decades. In our study, DEGs in GAs and ergosterol biosynthesis were all decreased at the YW3 stage compared to the YW1 stage, consistent with previous results that triterpenoid content was lower at the mature stage. Previous studies have also confirmed the antimicrobial [75], antifungal, and antiviral activities of some triterpenoids [76,77]. In addition, environmental factors also affect GAs biosynthesis [13], indicating that triterpenoids accumulated at the primordia might protect the organism from abiotic and biotic stress. Work for improving the production of GAs has been done by overexpressing GA biosynthetic genes [78,79], while the increased GA content was limited. Therefore, we speculated that the over-accumulation of GAs might affect normal organism growth. A recent study showed that fruiting bodies contain more specific triterpenoids [4]. We found that two fumagillin β-trans-bergamotene synthases encoding genes, *GL18506* and *GL24809*, which function in the branched pathway of farnesyl diphosphate and finally synthesize vitamin K2, were differentially expressed during sporulation. These results indicated that the reduction in known metabolites might result from inclining to branched pathways, and further research is required to identify more secondary metabolites and prove their pharmacological effects, which could make up for the low concentration of GAs.

## Figures and Tables

**Table 1:** Functional annotation of *G. lucidum* deduced proteins by sequence similarity search.

Annotated_Database	Annotated_Number		300 ≤ Length < 1000 ^b^		Length ≥ 1000	
	All	New-Isoform ^a^	All	New-Isoform	All	New-Isoform
COG_Annotation	3831	17	928	9	2886	8
GO_Annotation	5502	72	1639	33	3779	39
KEGG_Annotation	3709	44	1136	21	2512	23
KOG_Annotation	4985	29	1268	12	3685	17
Pfam_Annotation	7207	49	1992	17	5164	32
Swiss-Prot_Annotation	5721	37	1472	16	4202	21
eggNOG_Annotation	9183	123	2820	39	6254	84
NR_Annotation	12,093	373	4318	146	7513	225
All_Annotated	12,109	375	4327	147	7518	226

^a^ New-isoform, genes that were not found in the monokaryotic strain G.260125-1, were identified in the YW-1 strain; ^b^ 300 ≤ length < 1000: genes with transcript length (bp).

**Table 2:** The number of differentially expressed genes annotated in multiple public databases.

Annotated_Database	YW2/YW1	YW3/YW2	YW3/YW1
COG	183	115	429
GO	187	127	465
KEGG	87	57	205
KOG	139	105	369
NR	510	388	1300
Pfam	263	183	656
Swiss-Prot	212	142	507
eggNOG	352	256	885
Total	511	388	1302

## Data Availability

The data presented in this study are deposited into the Sequence Read Archive database (accession number: PRJNA704770).

## Supplementary Materials

The following are available online: https://www.mdpi.com/article/10.3390/genes12040504/s1. Table S1. Primers used for qRT-PCR analysis of selected genes. Table S2. Summary of Illumina transcriptome sequencing of *Ganoderma lucidum*. Table S3. List of new-isoforms identified in the YW-1 strain as compared to G.260125-1. Table S4. List of differentially expressed genes between developmental stages of *Ganoderma lucidum* basidiospores. Table S5. The identified transcriptional regulators in YW-1. Table S6. Summary of differentially expressed transcription regulators. Table S7. Summary of the GATA-binding elements on the promoters of the annotated DEGs. Table S8. Summary of the DEGs containing the GATA binding motif. Table S9. Summary of DEGs, which were predicted to participate in meiotic process.
